# Finite size effect induces stochastic gamma oscillation in inhibitory network with conduction delay

**DOI:** 10.1186/1471-2202-15-S1-P115

**Published:** 2014-07-21

**Authors:** Grégory Dumont, Georg Northoff, André Longtin

**Affiliations:** 1 Physics department, University of Ottawa, Canada; 2 Mind, Brain Imaging and Neuroethics, Royal Ottawa Healthcare, Institute of Mental Health Research, Ottawa, Canada; 3 Center for Neural Dynamics, University of Ottawa

## 

Cortical gamma frequency (30-100 Hz) are known to be associated with many cognitive processes. Understanding the dynamics in the gamma band is crucial in neuroscience. Stochastic gamma oscillations due to finite size effects were reported using the stochastic Wilson-Cowan model ([1] and [2]). On the other hand, temporal correlation can be induced by excitation [3] as well as inhibition [4]. Especially, oscillations induced by the conduction delay of the inhibitory feedback is a possible mechanism ([4], [5] and [6]) for gamma rhythms. We investigate the role of both finite size effects and the conduction delay on the emergence of gamma oscillations in an inhibitory all-to-all neural network. To this end, we expand the recently proposed linear noise approximation (LNA) technique to this non-markovian ”delay” case [1]. This allows us to compute a theoretical expression for the power spectrum of the population activity. Our analytical result is in good agreement with the power spectrum obtained via numerical simulations for a range of parameters.

**Figure 1:**
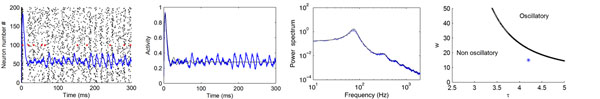
The left panel is a raster plot of 200 interneurons. The blue line represents the global activity of the network. The second panel shows a comparison between the stochastic process and its deterministic counterpart. The third panel compares the numerical (blue) and theoretical (black) power spectra. The last panel shows the phase diagram as a function of the feedback weight and the delay.

We show in the left part of the Figure 1 a raster plot depicting the spiking time of each neuron where each neuron follows the stochastic random walk between active and silent state, in red the spike timing of one particular neuron. The second plot is a comparison between the deterministic rate model (black curve) and the stochastic spiking process (blue curve). If the deterministic counterpart reaches a stable fixed point, the stochastic process exhibits self sustained oscillations. In the third plot, the theoretical power spectrum obtained via the LNA method (black curve) and the power spectrum obtained from the numerical simulations (blue curve) are shown to agree nicely. In the last plot we give the phase diagram, showing that the model is under the oscillatory regime. This tells us that gamma oscillations can be caused by the combination of delay and finite size effects in such an inhibitory neural network.
